# Radiative MHD Casson Nanofluid Flow with Activation energy and chemical reaction over past nonlinearly stretching surface through Entropy generation

**DOI:** 10.1038/s41598-020-61125-9

**Published:** 2020-03-10

**Authors:** Zahir Shah, Poom Kumam, Wejdan Deebani

**Affiliations:** 1 Center of Excellence in Theoretical and Computational Science (TaCS-CoE), SCL 802 Fixed Point Laboratory, Science Laboratory Building, King Mongkut’s University of Technology Thonburi (KMUTT), 126 Pracha-Uthit Road, Bang Mod, Thrung Khru, Bangkok 10140 Thailand; 2 KMUTT Fixed Point Research Laboratory, Room SCL 802 Fixed Point Laboratory, Science Laboratory Building, Department of Mathematics, Faculty of Science, King Mongkut’s University of Technology Thonburi (KMUTT), 126 Pracha-Uthit Road, Bang Mod, Thrung Khru, Bangkok 10140 Thailand; 3 Department of Medical Research, China Medical University Hospital, China Medical University, Taichung, 40402 Taiwan; 4 Department of Mathematics, College of Science & Arts, Rabigh King Abdul-Aziz University, Jeddah, 21911 Saudi Arabia

**Keywords:** Mechanical engineering, Applied mathematics, Nanoparticles, Fluid dynamics

## Abstract

In the present research analysis we have addressed comparative investigation of radiative electrically conducting Casson nanofluid. Nanofluid Flow is assumed over a nonlinearly stretching sheet. Heat transport analysis is carried via joule dissipation, thermal behavior and convective boundary condition. To employ the radiative effect radiation was involved to show the diverse states of nanoparticles. Furthermore entropy optimization with activation energy and chemical reaction are considered. Thermodynamics 2^nd^ law is applied to explore entropy generation rate. Nonlinear expression is simplified through similarity variables. The reduced ordinary system is tackled through optimal approach. Flow pattern was reported for wide range of scrutinized parameters. Computational consequences of velocity drag force, heat flux and concentration gradient are analyzed numerically in tables. Results verify that conduction mode augments with enhance of magnetic parameter.Increasing radiation boosts the temperature and entropy. Activation energy corresponds to augmented concentration. Heat transmission rate augments with the consideration of radiation source term.

## Introduction

The researcher has grown consideration in the non-Newtonian fluids due to their remarkable features in the area of technological and industrial sciences. For instance, synthetic lubricants, drilling muds, certain oils, paints, sugar solutions, clay coating, and biological fluid like blood are the just communal cases of non-Newtonian fluids. The Navier-Stokes fundamental equations cannot momentarily define the characteristics of the flow field of non-Newtonian fluids due to the complexity in the mathematical formulation of the flow problem. Abundant models for non-Newtonian fluids are defined as deliberate rheological qualities such as Eyring-Powell, Bulky, Seely, Oldroyd-B, Maxwell, Oldroyd-A, Carreau, Casson, Burger, Jeffrey, etc. Amongst these models, the most significant model for the blood properties and suspensions in our daily life is the Casson model^1^. For the purpose of developments in fluids study, considering Soret and Dufour effects, Hayat *et al*.^2^ proposed the flow of Casson fluid. The details research work about Casson fluid can be seen in^3–6^. Saravana *et al*.^7^ have been studied magnetohydrodynamic Casson fluid with inconstant thickness under the aligned magnetic field.

Due to its frequent uses in engineering and industrial, the theme of nanofluidic flow has attracted widespread attention over the past two decades. Researchers and scientists have not only discovered the astonishing thermal properties of nanofluids, but improving the causes of thermal conductivity of nanofluids are also planned, the integration of biotechnological mechanisms and nanofluids may provide capable applications in biosensors, agriculture and pharmaceuticals. In the biotechnology field, there are many nanomaterials in practice, such as nanofibers, nanoparticles, nanostructures and nanowires. Nanobiotechnology has a reliable market and it is predictable that the future is very bright of such products. Similarly, the importance of nanofluids and microfluidics is undeniable in the field of procedures and biomedical devices. Magnetic nanofluids have both liquid and magnetic properties; it has many applications such as tunable fiber filters, modulators, optical switches and gratings. In medicine, cancer treatment, sink float separation and speaker magnetic nanoparticles play a vital role. The main source of renewable energy is solar energy is and has the least ecological pollution. A person can get electricity, water and energy directly from a solar source. Researchers believe that the solar collection process can be triggered by inserting nanoparticles into the fluid. In several industrial processes cooling and heating of fluids are major requirements such as power manufacturing and delivery. Every day, there is a need to improve the cooling process of high-energy equipment^8–11^. Zubair *et al*.^12^ presented the MHD Casson nanofluid flow with entropy generation. Saleem *et al*.^13,14^ have investigated radiated magneto nanomaterial with Convective heat and mass transfer using viscous dissipation and source of heat source. Some recent study about nanofluid in different geometries with diverse properties can be reads in^15–21^.

The procedure of heat transmission in engineering and scientific processes is exceedingly reliant on structure of the surface from which heat transfer occurs to the fluid. The phenomenon of heat transfer occurs due to temperature differences. Heat exchanger is the foremost thermal device used to transfer heat and widely used in many types of thermal applications. Mainly it is uses in energy resources of universe by application of energy that could only be achieved by heat transfer augmentation of heat exchanger. Hussanan *et al*.^22–24^ have investigated the heat transfer phenomena in convective form using different type of nanoparticle in different geometries. Saleem *et al*.^25^ study of Cattaneo-Christov heat flux model and heat transfer in nanofluid. The most recent investigation of heat transfer and nanofluid can be studied in^26–32^.

Entropy enhancement is utilized to clarify the presentation of different frameworks in modern and building applications. Hence as of late various researchers and engineers have concentrated they’re focused on entropy streamlining issues. Entropy is imitative from Greek word entropia, which implies that “a moving in the direction of” or “change”. Entropy calculation of flow and heat transfer systems is important as it classifies the factors which are responsible for the loss of useful energy. The loss of energy can take the effectiveness of the thermally designed system. By diminishing the factors that generate entropy the production of the system can be increased. Primarily, Bejan^33^ investigated entropy optimization problem. Ellahi *et al*.^34^ and Rashidi *et al*.^35^ investigated the entropy for nanofluid flow with convective heat transfer in different geometries. Atlas *et al*.^36^ examined entropy analysis in Casson nanofluid with of Cattaneo-Christov heat and mass flux model. Abolbashari *et al*.^37^ examined the entropy analysis in electrically conducting nanofluid, curving over lengthening sheet. Sheikholeslami *et al*.^38^ scrutinized entropy creation in magnetite nanofluid flow lattice Boltzmann approach. Some other important research about entropy optimization in nanofluid can be studied in^39–50^.

Above literature analysis shows the nonappearance of detailed of radiative electrically conducting Casson nanofluid with activation energy, entropy analysis and chemical reaction over a nonlinearly stretching sheet. Main goal of current article is to scrutinize nanofluid with activation energy and entropy optimization. Nonlinear expression is simplified through similarity variables. The reduced ordinary system is tackled through optimal approach. The source term of radiation impact is accounted for different states of nanoparticles. Behaviors of all scrutinized variables on hydrothermal behavior were demonstrated graphically. Results for heat and mass transfer rate are also calculated through tables.Performances of numerous engineering parameters on velocity, concentration, entropy generation and temperature are discussed.

## Problem Formulation

**Figure 1:**
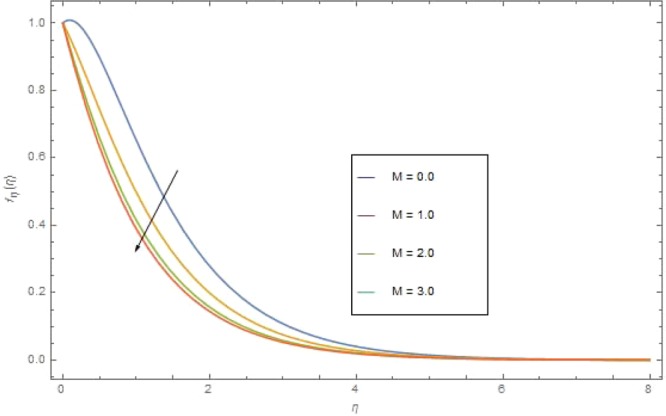
$${f}_{\eta }(\eta )$$ against M at $$\beta =0.4$$.

We assumed two dimensional electrically conducting thermally radiative steady Casson nanofluid flow through a past non-linear stretching surface. Two equal and opposite forces are applied to stretch the surface along x-direction (Fig. 1). The exponential velocity is defined as ${u}_{w}=a{x}^{{m}_{1}}$. The magnetic field *B*_*o*_ is applied normally to the stretching sheet where the electric filed for low magnetic Reynolds number $(Rm\ll 1)$ is not considered. Energy presentation is demonstrated in existence of chemical reaction, activation energy, Joule heating and thermal flux. Furthermore, Ludwig-Soret effect of the nanoparticles is considered. Entropy analysis is taken and for it Thermodynamic second mechanism is utilized to explore. The governing equations can be written as^1,5,17,49^

1 $$\frac{\partial v}{\partial y}+\frac{\partial u}{\partial x}=0$$

2 $$\frac{{\mu }_{nf}}{{\rho }_{nf}}\left(1+\frac{1}{\beta }\right)\left(\frac{{\partial }^{2}u}{\partial {x}^{2}}+\frac{{\partial }^{2}u}{\partial {y}^{2}}\right)-\frac{1}{{\rho }_{nf}}{\sigma }_{nf}{B}_{0}^{2}u=v\frac{\partial u}{\partial y}+u\frac{\partial u}{\partial x},$$

3 $$\begin{array}{ccc}\left(u\frac{\partial {\rm{{\rm T}}}}{\partial x}+v\frac{\partial {\rm{{\rm T}}}}{\partial y}\right)+\frac{1}{{(\rho {C}_{p})}_{nf}}\frac{\partial {q}_{r}}{\partial y}-\frac{\sigma {B}_{o}^{2}}{{(\rho {C}_{p})}_{nf}}{u}^{2} & = & {k}_{nf}{(\rho {c}_{p})}_{nf}^{-1}\left(\frac{{\partial }^{2}{\rm{{\rm T}}}}{\partial {y}^{2}}+\frac{{\partial }^{2}{\rm{{\rm T}}}}{\partial {x}^{2}}\right)\\  &  & +\frac{{(\rho {c}_{p})}_{nf}}{{(\rho {c}_{p})}_{f}}\left[{D}_{B}\left(\frac{\partial {\rm{{\rm T}}}}{\partial y}\frac{\partial C}{\partial y}\right)+\frac{{D}_{T}}{{T}_{\infty }}{\left(\frac{\partial {\rm{{\rm T}}}}{\partial y}\right)}^{2}\right],\\  &  & \left[{{\rm{{\rm T}}}}^{4}\cong 4{{\rm{{\rm T}}}}_{c}^{3}{\rm{{\rm T}}}-3{{\rm{{\rm T}}}}_{c}^{4},{q}_{r}=-\frac{4{\sigma }_{e}}{3{\kappa }_{R}}\frac{\partial {{{\rm{{\rm T}}}}_{\infty }}^{4}}{\partial y}\right].\end{array}$$

4 $$\left(u\frac{\partial C}{\partial x}+v\frac{\partial C}{\partial y}\right)+{K}_{r}^{2}(C-{C}_{\infty }){\left(\frac{{\rm{{\rm T}}}}{{{\rm{{\rm T}}}}_{\infty }}\right)}^{{n}_{o}}{e}^{\left(\frac{-{{\rm E}}_{a}}{\kappa v}\right)}={D}_{B}\left(\frac{{\partial }^{2}C}{\partial {y}^{2}}\right)+\frac{{D}_{{\rm{{\rm T}}}}}{{{\rm{{\rm T}}}}_{\infty }}(\frac{{\partial }^{2}{\rm{{\rm T}}}}{\partial {y}^{2}}).$$

The applied boundary layer constraints are

5 $$\begin{array}{c}u=a{x}^{{\rm{M}}},\,\,\,\,\,v=0,\,\,\,\,\,\,\,\frac{\partial {\rm{{\rm T}}}}{\partial y}=\frac{h}{k}({\rm{{\rm T}}}-{{\rm{{\rm T}}}}_{f}),\,\,\,\\ \frac{\partial {\rm{{\rm T}}}{D}_{{\rm{{\rm T}}}}}{\partial y{{\rm{{\rm T}}}}_{\infty }}+\frac{\partial C}{\partial y}{D}_{B}=0\,\,\,\,\,at\,\,\,\,\,y=0,\\ u\to 0,\,C\to {C}_{\infty },\,{\rm{{\rm T}}}\to {{\rm{{\rm T}}}}_{\infty }\,\,\,\,\,as\,\,\,\,\,y\to \infty .\end{array}$$

The velocity profile is $(u,v,0)$ in the directions of the $(x,\,y)$,where in $\mu ={\rho }_{nf}\upsilon ,$ $\upsilon ,\,\mu$ represents kinematic and viscosities, ${\rho }_{f}$ show nanofluid density, ${\sigma }_{nf}$ is conductivity of nanofluid due to electric current and *B* represents the magnetic force. In Eq. (4), T is the temperature of the fluid, in relation $\alpha {(\rho c)}_{f}=\kappa$, $\alpha$ use for thermal diffusivity, $\kappa$ for thermal conductivity, *c* represents heat capacity. In relation ${q}_{r}=-\frac{4{\sigma }_{e}}{3{\kappa }_{R}}\frac{\partial {{\rm T}}^{4}}{\partial y}$, ${q}_{r}$ is represents radiative heat flux, ${\kappa }_{R}$ is mean absorption coefficient and ${\sigma }_{e}$ is the Stefan-Boltzman. The terms ${D}_{B},\,{D}_{T}$ signifies Brownian and thermophoretic individually. In Eq. (5), *C* represents concentration, the relation $\frac{\delta }{{\delta }_{t}}={x}^{\frac{{\rm{M}}-1}{2}}$ represents mutable heat transmission coefficient, ${K}_{r}$ is the rate of chemical reaction rate, ${{\rm E}}_{\alpha }$ is the activation energy, ${n}_{o}$ is fitted rate constant.

Considering the similarity transformation^49^

6 $$\begin{array}{c}u=a{x}^{{\rm{M}}}{f}_{\eta }(\eta ),\\ v=-{\left(\frac{a({\rm{M}}+1)v}{2}\right)}^{\frac{1}{2}}\left(f(\eta )+\eta \frac{{\rm{M}}-1}{{\rm{M}}+1}{f}_{\eta }(\eta )\right){x}^{\frac{{\rm{M}}-1}{2}}\\ \theta (\eta )=\frac{{\rm{{\rm T}}}-{{\rm{{\rm T}}}}_{\infty }}{{{\rm{{\rm T}}}}_{f}-{{\rm{{\rm T}}}}_{\infty }},\,\phi (\eta )=\frac{C-{C}_{\infty }}{{C}_{\infty }},\\ {\rm{Where}}\,\eta ={x}^{\frac{{\rm{M}}-1}{2}{\left(\frac{a({\rm{M}}+1)}{2v}\right)}^{\frac{1}{2}}y}\end{array}\}$$

Using Eq. (6) in Eqs. (1–5) we obtained

7 $${f}_{\eta \eta \eta }+f{f}_{\eta \eta }-\left(1+\frac{1}{\beta }\right){({f}_{\eta })}^{2}\left(\frac{2{\rm{M}}}{{\rm{M}}+1}\right)-{M}^{2}\left(\frac{2}{{\rm{M}}+1}\right){f}_{\eta }=0$$

8 $${\theta }_{\eta \eta }(1+{N}_{r})+\Pr \,f\theta +\Pr ({{\rm{{\rm N}}}}_{t}{{\theta }_{\eta }}^{2}+{{\rm{{\rm N}}}}_{b}{\theta }_{\eta }{\phi }_{\eta })+\Pr {{\rm{{\rm M}}}}^{2}{E}_{c}\left(\frac{2}{{\rm{M}}+1}\right){({f}_{\eta })}^{2}=0,$$

9 $${\phi }_{\eta \eta }+Sc{\phi }_{\eta }f+\left(\frac{{N}_{t}}{{N}_{b}}\right){\theta }_{\eta \eta }-{(1+\theta \delta )}^{{n}_{o}}{e}^{\left(\frac{-E}{1+\theta \delta }\right)\phi }Sc{\sigma }_{1}\left(\frac{2}{{\rm{M}}+1}\right)=0.$$

10 $$\begin{array}{c}{f}_{\eta }(\eta )=1,\,f(\eta )=0,\,Nb{\phi }_{\zeta }(\eta )+Nt{\theta }_{\eta }(\eta )=0\,\,at\,\,\eta =0,\\ \,\,{\theta }_{\eta }(\eta )={\gamma }_{1}[\theta (\eta )-1],\,{f}_{\eta }(\eta )\to 0,\,\phi (\eta )\to 0,\,\theta (\eta )\to 0\,\,as\,\,\eta \to \infty \end{array}$$

The modeled parameters after sampling the governing equations are obtained as

11 $$\begin{array}{rcl}{\rm M} & = & {\left(\frac{\sigma {B}_{o}^{2}{x}^{1-{m}_{1}}}{a{\rho }_{f}}\right)}^{\frac{1}{2}},\,{N}_{r}=\frac{16{\sigma }^{\ast }{{\rm T}}_{r}^{3}}{3\kappa {k}^{\ast \ast }},\,{P}_{r}=\frac{\nu }{\alpha },{{\rm N}}_{b}=\frac{{D}_{B}{\tau }^{\ast }{C}_{\infty }}{\overrightarrow{v}},\\ {{\rm N}}_{t} & = & \frac{{D}_{T}{\tau }^{\ast }[{{\rm T}}_{f}-{{\rm T}}_{\infty }]}{\overrightarrow{v}{{\rm T}}_{\infty }},Ec=\frac{{(a{x}^{{m}_{1}})}^{2}}{{c}_{p}({{\rm T}}_{f}-{{\rm T}}_{\infty })},Sc=\frac{\nu }{{D}_{B}},{\sigma }_{1}=\frac{{K}_{r}^{2}{x}^{1-{m}_{1}}}{a},E=\frac{{E}_{\alpha }}{{k}_{B}{{\rm T}}_{\infty }}\end{array}$$

Here ${\rm{{\rm M}}},{N}_{r},{{\rm{P}}}_{r},{{\rm N}}_{b},{{\rm{{\rm N}}}}_{t},Ec,Sc,{\sigma }_{1},E$ magnetic parameter, parameter of thermal radiation, Prandtl number, Brownian motion parameter, thermophoresis parameter, Eckert number, Schmidt number, reaction rate constant, dimensionless activation energy and the thermal Biot number.

## Entropy Generation and Modeling

The entropy generation is mathematically expressed as

12 $${S}_{G}=\frac{k}{{T}_{\infty }^{2}}{({{\rm{{\rm T}}}}_{y})}^{2}\left(\frac{16{\sigma }^{\ast }{T}_{\infty }^{3}}{3k{k}^{\ast \ast }}+1\right)+\frac{\sigma {B}_{o}^{2}}{{T}_{\infty }}{\overrightarrow{u}}^{2}+\left(\frac{RD}{{C}_{\infty }}{({C}_{y})}^{2}+\frac{RD}{{T}_{\infty }}({C}_{y}{{\rm{{\rm T}}}}_{y})\right)$$

Which after simplification give the form

13 $${N}_{G}=({{\rm{{\rm N}}}}_{r}+1)\left(\frac{{\rm{M}}+1}{2}\right){({\theta }_{\eta })}^{2}{\alpha }_{1}+{{\rm M}}^{2}{B}_{r}{({f}_{\eta })}^{2}+\frac{({\rm{M}}+1)}{2}\frac{L}{{\alpha }_{1}}{({\phi }_{\eta })}^{2}+\frac{({\rm{M}}+1)}{2}L{\theta }_{\eta }{\phi }_{\eta }$$

Here

14 $${{\rm{{\rm N}}}}_{G}=\frac{\nu {S}_{G}{{\rm T}}_{\infty }}{\alpha \kappa ({{\rm T}}_{f}-{{\rm T}}_{\infty }){x}^{{\rm{M}}-1}},{\alpha }_{1}=\frac{{{\rm{{\rm T}}}}_{f}-{{\rm{{\rm T}}}}_{\infty }}{{{\rm{{\rm T}}}}_{\infty }},Br=\frac{\mu {a}^{2}{x}^{2{\rm{M}}}}{\kappa ({{\rm{{\rm T}}}}_{f}-{{\rm{{\rm T}}}}_{\infty })},L=\frac{{R}_{D}{C}_{\infty }}{\kappa }.$$

${{\rm{{\rm N}}}}_{G},{\alpha }_{1}$, *L* and *Br* indicates the rate of entropy optimization rate, gradient of temperature, Brinkman number and diffusive variable respectively.

## Physical Quantities

### Surface drag force

The physical quantities Skin friction coefficients ${C}_{Fx}$ is defined as

15 $${C}_{Fx}=\frac{2{\varphi }_{w}}{\rho {u}_{w}^{2}},$$

Shear stress for the Casson fluid is defined as

16 $${\varphi }_{w}={\left[\mu {u}_{y}+(1+\frac{1}{\beta }){u}_{y}\right]}_{y=0},$$

The dimensionless form is

17 $${\mathrm{Re}}_{x}^{1/2}{C}_{Fx}=\sqrt{\frac{{\rm{M}}+1}{2}}(1+\frac{1}{\beta }){f}_{\eta \eta }(0).$$

In which ${\mathrm{Re}}_{x}^{1/2}$ indicates the local Reynold number.

### Heat transfer rate

The Local Nusselt number or temperature gradient $N{u}_{x}$ is

18 $${\rm{{\rm N}}}{u}_{x}=\frac{x{Q}_{w}}{k({{\rm T}}_{f}-{{\rm T}}_{\infty })},$$

Heat flux ${Q}_{w}$ is defined as

19 $${{Q}_{w}=-\kappa \left(\frac{4{\sigma }_{e}}{3\kappa {\kappa }_{R}}{{\rm{{\rm T}}}}_{\infty }^{3}{}_{y}+1\right){{\rm{{\rm T}}}}_{y}|}_{y=0},$$

The dimensionless form is

20 $${\mathrm{Re}}_{x}^{-1/2}N{u}_{x}=-\sqrt{\frac{{\rm{M}}+1}{2}}{\theta }_{\eta }(0).$$

### Mass transfer rate

Sherwood number $S{h}_{x}$ are stated as

21 $${S}_{hx}=\frac{x{h}_{w}}{{D}_{B}({C}_{f}-{C}_{\infty })},$$

22 $${{J}_{w}=-{D}_{B}{C}_{y}|}_{y=0}$$

The dimensionless form is

23 $${\mathrm{Re}}_{x}^{-1/2}S{h}_{x}=-\sqrt{\frac{{\rm{M}}+1}{2}}{\phi }_{\eta }(0)$$

## Solution via HAM

Here we have used optimal approach to get computational results. Due to couple nonlinear system of governing differential equations system homotopy analysis scheme is proposed to compute the solutions. Homotopy analysis scheme is independent of small or large parameters and needs no discretization. This scheme has no stability issues like seen in numerical approaches. This scheme needs the choice of linear operator and initial guess. Initial guess is selected in such a manner that it satisfies the given boundary conditions. Initial guesses are

24 $${F}_{0}(\eta )=\frac{{e}^{\eta }-1}{{e}^{\eta }},\,{\Theta }_{0}(\eta )=\frac{{\beta }_{1}}{1+{\beta }_{1}}{e}^{-\eta },{\Phi }_{0}(\eta )=\frac{Nt}{Nb}\frac{1}{1+{\beta }_{1}}{e}^{-\eta }.$$

The corresponding to linear operators are

25 $${L}_{F}(F)={F}_{\eta \eta \eta }-{F}_{\eta },\,{{\rm{L}}}_{\hat{\Theta }}{(\Theta )=\Theta }_{\eta \eta }-\Theta ,{{\rm{L}}}_{\varPhi }(\Phi )={\Phi }_{\eta }-\Phi .$$

these linear operators conform following features

26 $$\begin{array}{c}{L}_{F}({{\rm E}}_{1}+{{\rm E}}_{2}{e}^{-\eta }+{{\rm E}}_{3}{e}^{\eta })=0,\\ {{\rm{L}}}_{\Theta }({{\rm E}}_{4}{e}^{-\eta }+{{\rm E}}_{5}{e}^{\eta })=0,\\ {{\rm{L}}}_{\Phi }({{\rm E}}_{6}{e}^{-\eta }+{{\rm E}}_{7}{e}^{\eta })=0.\end{array}\}$$

Here $\mathop{\sum }\limits_{n=1}^{7}{{\rm E}}_{n},$ with $n=1,2,3\ldots$ are subjective constants.

## Discussion

The steady radiative electrically conducting Casson nanofluid flow through a nonlinearly stretching sheet is investigated with joule dissipation, thermal behavior and convective boundary condition. Optimal approach is used due to couple nonlinear system of governing differential equations system to get computational results. Momentous features of various intriguing parameters on entropy, velocity, concentration and temperature are deliberated through graphs. Surface drag force, temperature gradient and mass transmission rate are numerically intended versus various engineering variables.

### Velocity

**Figure 2:**
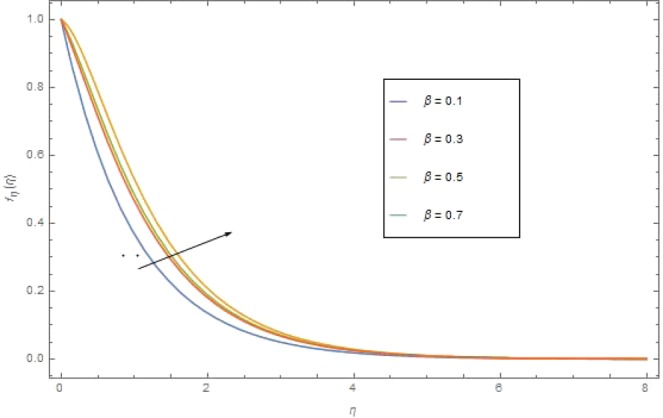
$${f}_{\eta }(\eta )$$ against $$\beta $$ at $${\rm{{\rm M}}}=1.0$$.

Figure 1 presented the significant effect of M on ${f}_{\eta }(\eta )$. Inverse variation is seen between M and ${f}_{\eta }(\eta )$. For greater value of (M) the Lorentz forces enhances which raises the resistive force to the nanofluid motion and in result the velocity ${f}_{\eta }(\eta )$ reduces. Effect of the Casson parameter *β* on ${f}_{\eta }(\eta )$ is depicted in Fig. 2. Increasing performance is perceived in ${f}_{\eta }(\eta )$ for higher value of *β*. Casson parameter *β* is allied with the non-Newtonian Casson fluid nature and it has inverse variation to the yield stress. Therefore, enhancing *β* reduces the shear stress of the fluid and in turn it relaxes the fluid to move with higher velocity. This effect is very clear in Fig. 2.

### Temperature

**Figure 3:**
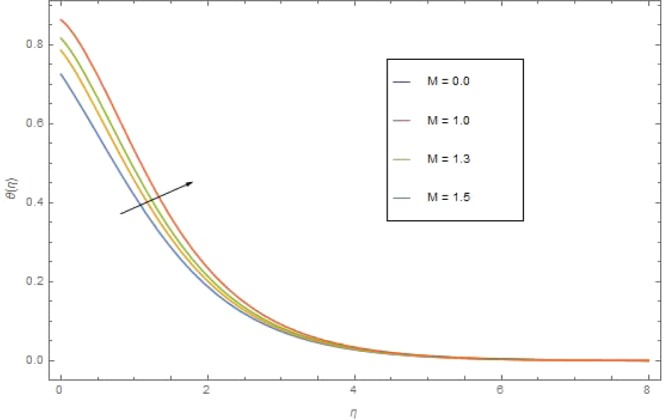
$$\theta (\eta )$$ against M when $${N}_{r}=0.5,\,{{\rm{P}}}_{r}=2.5,\,{{\rm{{\rm N}}}}_{b}={{\rm{{\rm N}}}}_{t}=0.4,\,{E}_{c}=0.7$$.

**Figure 4:**
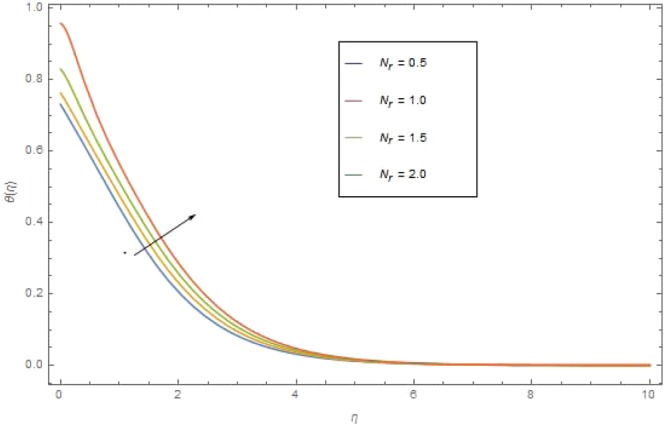
$$\theta (\eta )$$ against *N*_*r*_ when $${\rm{{\rm M}}}=0.5,{{\rm{P}}}_{r}=2.5,{{\rm{{\rm N}}}}_{b}={{\rm{{\rm N}}}}_{t}=0.4,\,{E}_{c}=0.7$$.

**Figure 5:**
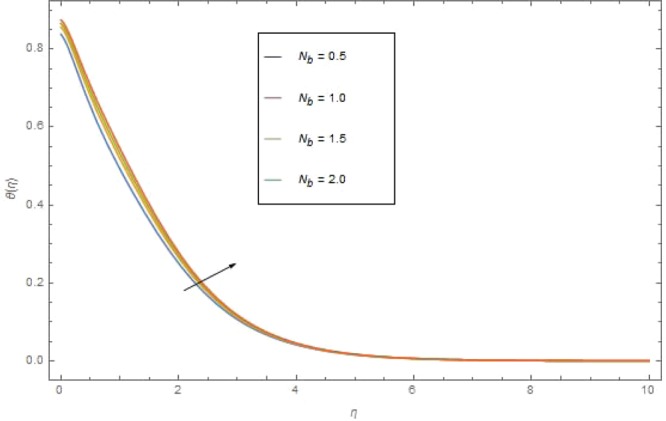
$$\theta (\eta )$$ against *N*_*r*_ when $${\rm{{\rm M}}}=1.5,\,{{\rm{P}}}_{r}=1.5,\,{N}_{r}=0.3,\,{{\rm{{\rm N}}}}_{t}=0.4,\,{E}_{c}=0.6$$.

**Figure 6:**
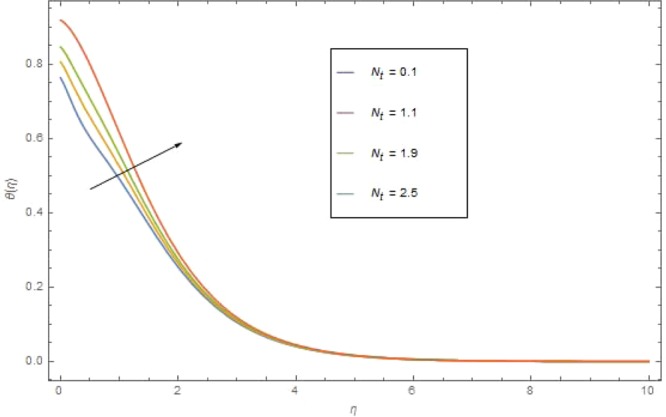
$$\theta (\eta )$$ against *N*_*t*_ when $${\rm M}={N}_{r}=1.5,\,{N}_{b}=0.7,\,{{\rm P}}_{r}=6.4,\,{E}_{c}=1.6$$.

**Figure 7:**
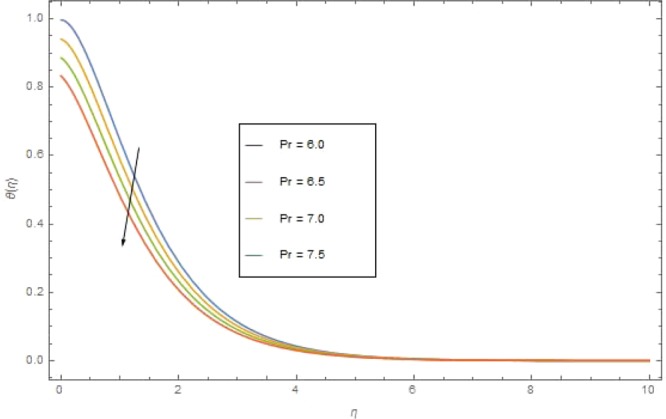
$$\theta (\eta )$$ against P_*r*_ when $${\rm{{\rm M}}}={N}_{r}=0.5,\,{N}_{r}=0.3,\,{{\rm{{\rm N}}}}_{t}=0.4,\,{E}_{c}=0.6$$.

**Figure 8:**
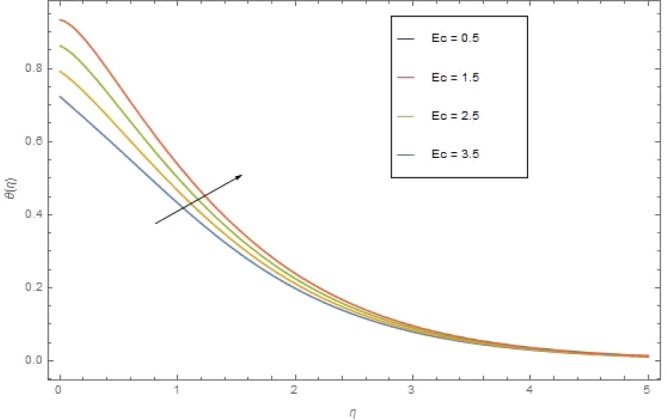
$$\theta (\eta )$$ against E_*c*_ when $${\rm{{\rm M}}}={N}_{r}={N}_{t}=0.9,\,{{\rm{{\rm P}}}}_{r}=6.4,\,{E}_{c}=0.6$$.

The significant effects of various parameters modelled from temperature Eq. (3) like $({\rm{{\rm M}}}),({N}_{r}),({{\rm{{\rm N}}}}_{b}),({{\rm{{\rm N}}}}_{t}),({{\rm{P}}}_{r})$ and $({E}_{c})$ on $\theta (\eta )$ are shown in Figs. (3–8). Figure 3 is present the impact of *M* on $\theta (\eta )$. The augmented values of *M* augmented the heat transfer rate and temperature profile $\theta (\eta )$ increases. Apparently for higher (M) the Lorentz forces increases which augments the opposing forces to the fluid particles and hence the temperature enhances. Figure 4 presented the impact of a very imperative parameter ${N}_{r}$ on temperature profile $\theta (\eta )$. Physically $({N}_{r})$ is the relative involvement of heat transmission conduction to thermal radiation transfer. We observed enhancement in temperature field $\theta (\eta )$ with higher value of radiation parameter $({N}_{r})$. Augmentation in $({N}_{r})$ generates more heat which turn increases the nanofluid temperature. The impacts parameter $({{\rm{{\rm N}}}}_{b})$ and parameter $({{\rm{{\rm N}}}}_{t})$ on the $\theta (\eta )$ are elucidated in Figs. 5 and 6. Enhancing $({{\rm{{\rm N}}}}_{b})$ leads to the faster random motion of nanoparticles in fluid flow which displays an extension in thermal boundary layer thickness and augments the temperature of nanofluid more rapidly. A similar configuration is perceived for growing values (Nt) on $\theta (\eta )$. As in procedure of thermophoresis, more heated particles near the surface travel away from heated regions toward the cold region and rise temperature there and collective temperature of the whole system rises. Effect of $({P}_{r})$ on $\theta (\eta )$ is presented in Fig. 7. Clearly temperature is a decreasing function of $({P}_{r})$. Relation between $({E}_{c})$ Eckert number and temperature function $\theta (\eta )$ is shown in Fig. 8. Eckert number is relation amongst the variance boundary layer enthalpy and flows of kinetic energy, which described the transmission dissipation. Increasing $({E}_{c})$ augmented the internal energy of nanofluid which in turn, augmented the heat transfer rate.

### Concentration

**Figure 9:**
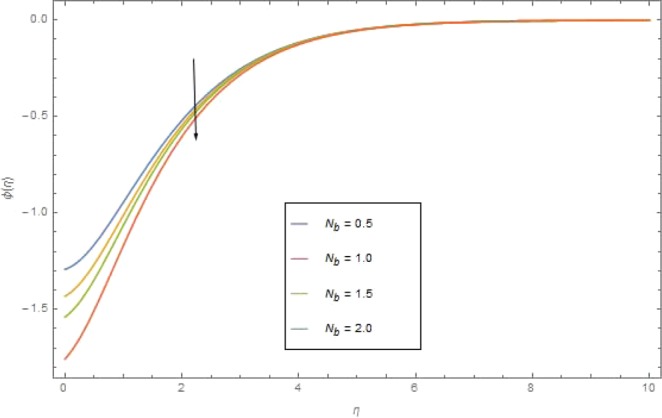
$$\phi (\eta )$$ against $${N}_{r}$$ when $$Sc=1.3,\,{\rm E}=0.5,\,{\sigma }_{1}=0.6,\,{\rm{{\rm M}}}=1.5,\,{{\rm{P}}}_{r}=1.5,\,{N}_{r}=0.3,\,{{\rm{{\rm N}}}}_{t}=0.4,$$$${E}_{c}=0.6$$.

**Figure 10:**
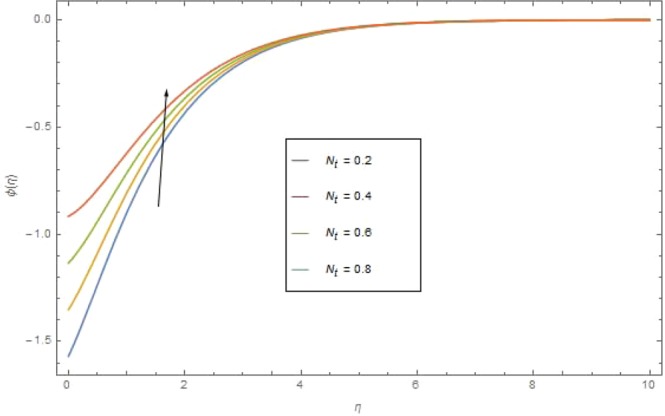
$$\phi (\eta )$$ against $${N}_{t}$$ when $$Sc={\rm E}=1.3,\,={\sigma }_{1}=0.6,\,\,{\rm{{\rm M}}}={N}_{r}=1.5,\,{N}_{b}=0.7,\,{{\rm{{\rm P}}}}_{r}=6.4,\,{E}_{c}=1.6$$.

**Figure 11:**
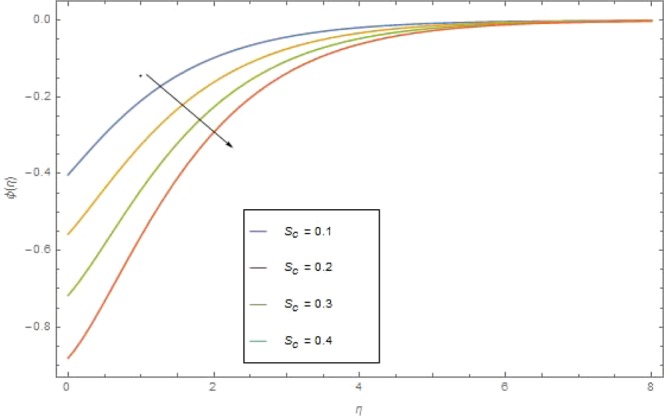
$$\phi (\eta )$$ against $${S}_{c}$$ when $${\rm E}=1.3,\,={\sigma }_{1}=0.6,\,{\rm{{\rm M}}}={N}_{r}=1.5,\,{N}_{b}={N}_{t}=0.7,\,{{\rm{{\rm P}}}}_{r}=6.4,\,{E}_{c}=1.6$$.

**Figure 12:**
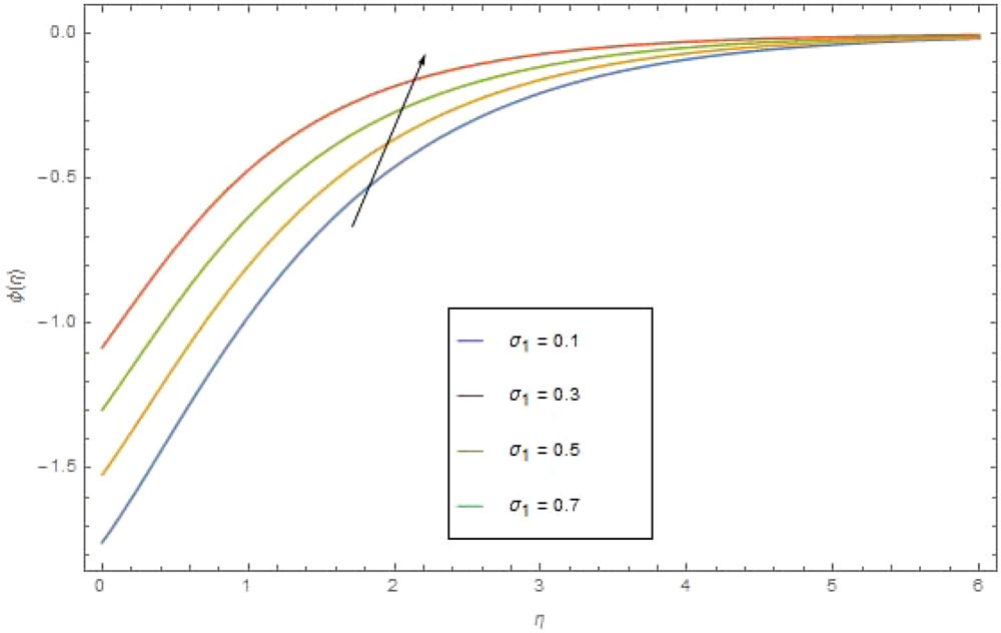
$$\phi (\eta )$$against $${\sigma }_{1}$$ when $${N}_{b}={\rm E}=0.7,\,{\rm{{\rm M}}}={N}_{r}=1.5,\,{N}_{b}=0.7,\,{{\rm{{\rm P}}}}_{r}=6.4,\,{E}_{c}=1.6$$.

**Figure 13:**
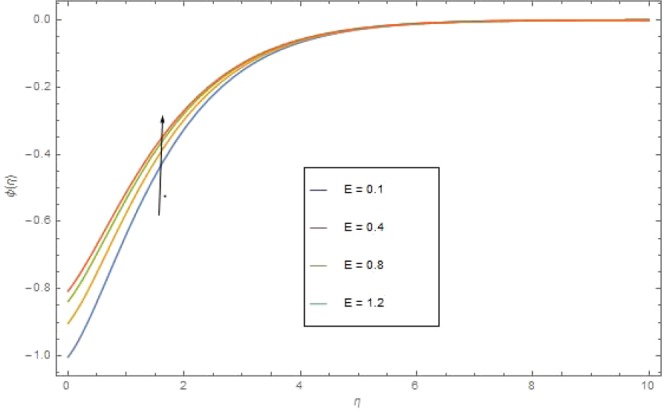
$$\phi (\eta )$$ against $${\rm E}$$ when $${\rm{{\rm M}}}={\sigma }_{1}=0.9,\,{N}_{r}=1.5,\,{N}_{b}={N}_{t}=0.7,\,{{\rm{{\rm P}}}}_{r}=6.4,\,{E}_{c}=1.6$$.

The significant effects of various parameters modelled from concentration Eq. (4) like $({{\rm{{\rm N}}}}_{b}),({{\rm{{\rm N}}}}_{t}),(Sc),({\sigma }_{1})$ and $(E)$ on concentration profile are shown in Figs. (9–13). The influences of parameter (N_*b*_) and parameter (N_*t*_) on the concentration profile $\phi (\eta )$ are elucidated in Figs. 9 and 10. Increasing value of (N_*b*_) reduces $\phi (\eta )$ while increasing (N_*t*_) augmented $\phi (\eta )$. Boosting (N_*t*_) enhances the motion of nanoparticles from higher to lower temperature gradient which in turn, exploit the concentration of nanoparticles. Figure 11 is drawn to scrutinize the behavior of $(Sc)$ on $\phi (\eta )$. Increasing $(Sc)$ the mass diffusivity decays and thus concentration is declined. Figure 12 is sketch to examine the behavior of reaction rate $({\sigma }_{1})$ on $\phi (\eta )$. It is observed that concentration $\phi (\eta )$ is increasing function for $({\sigma }_{1})$. The impact of activation energy parameter $(E)$ on concentration $\phi (\eta )$ is presented in Fig. 13. The higher value of activation energy augmented the concentration $\phi (\eta )$ of nanofluid.

### Entropy

**Figure 14:**
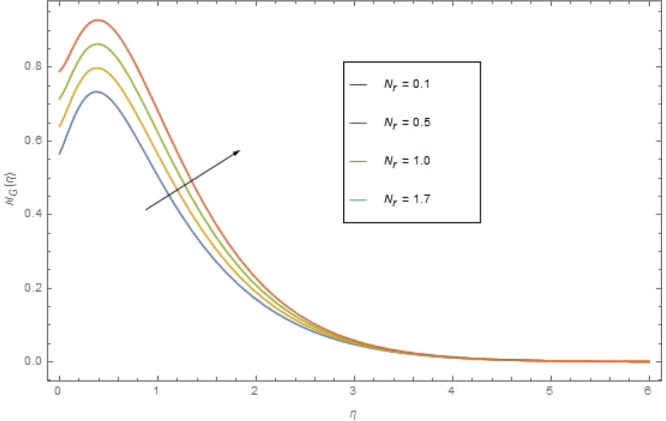
$${{\rm N}}_{G}(\eta )$$ against $${{\rm N}}_{r}$$ when $${{\rm B}}_{r}=1.2,\,{\alpha }_{1}=0.3,{\rm{{\rm M}}}=1,\,{\sigma }_{1}=0.4,\,{N}_{b}={N}_{t}=0.7,\,{{\rm{{\rm P}}}}_{r}=6.4,\,{E}_{c}=0.4$$.

**Figure 15:**
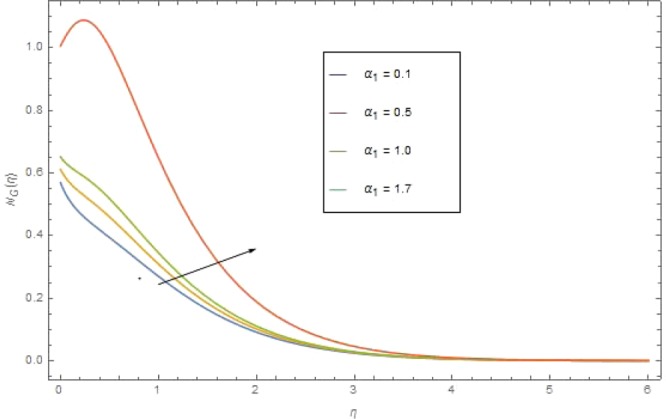
$${{\rm N}}_{G}(\eta )$$ against $${\alpha }_{1}$$ when $${{\rm{{\rm B}}}}_{r}=1.2,\,{{\rm{{\rm N}}}}_{r}={\rm{{\rm M}}}=1.5,\,{\sigma }_{1}=0.4,\,{N}_{b}={N}_{t}=0.7,\,{{\rm{{\rm P}}}}_{r}=6.4,\,{E}_{c}=0.4$$.

**Figure 16:**
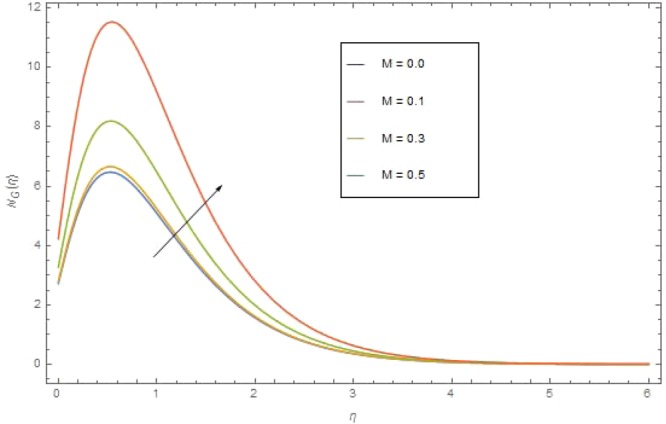
$${{\rm{{\rm N}}}}_{G}(\eta )$$ against M when $${{\rm{{\rm B}}}}_{r}=1.2,\,{\alpha }_{1}=0.3,\,{{\rm{{\rm N}}}}_{r}=1,{\sigma }_{1}=0.4,\,{N}_{b}={N}_{t}=0.7,\,{{\rm{{\rm P}}}}_{r}=6.4,\,{E}_{c}=0.4$$.

**Figure 17:**
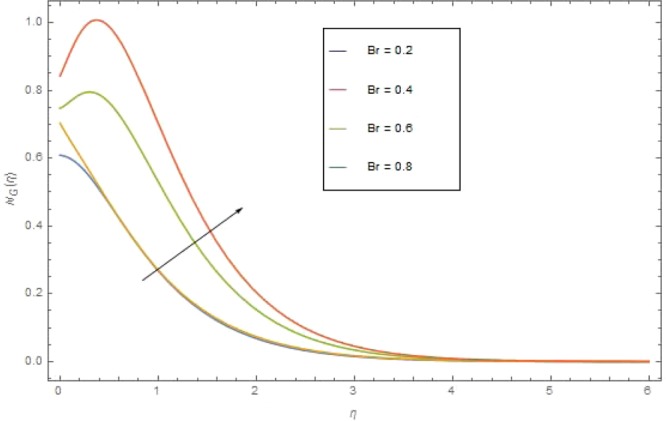
$${{\rm N}}_{G}(\eta )$$against $${{\rm B}}_{r}$$ when $${N}_{r}=1.2,\,{\alpha }_{1}=0.3,\,{\rm{{\rm M}}}=1,\,{\sigma }_{1}=0.4,\,{N}_{b}={N}_{t}=0.7,\,{{\rm{{\rm P}}}}_{r}=6.4,\,{E}_{c}=0.4$$.

The significant effects of various parameters modelled from concentration Eq. (12) like $({{\rm{{\rm N}}}}_{r}),({\alpha }_{1}),({\rm{{\rm M}}})$ and $({{\rm{{\rm B}}}}_{r})$ on entropy profile ${{\rm{{\rm N}}}}_{G}(\eta )$ are shown in Figs (14–17). Fig. 14 illustrates the variation of radiation parameter $({{\rm{{\rm N}}}}_{r})$ on ${{\rm{{\rm N}}}}_{G}(\eta )$. Increasing $({{\rm{{\rm N}}}}_{r})$ increases the emission of thermal radiation and as a results entropy ${{\rm{{\rm N}}}}_{G}(\eta )$ of the nanofluid augmented. The Significant effects of $({\alpha }_{1}),({\rm{{\rm M}}})$ and $({{\rm{{\rm B}}}}_{r})$ on ${{\rm{{\rm N}}}}_{G}(\eta )$ are illustrates in Figs. (15–17). For higher value of these parameters $({\alpha }_{1}),\,({\rm{{\rm M}}})$ and $({{\rm{{\rm B}}}}_{r})$ the entropy ${{\rm{{\rm N}}}}_{G}(\eta )$ is found is found as increasing function. In Fig. 17 the influence of Brinkman number $({{\rm{{\rm B}}}}_{r})$ is described. Actually Brinkman number is a heat generated source within the fluid moving region. The heat generated together with the heat transfer from the wall increases the entropy optimization.

### Surface drag force, heat and mass transfer rate

Prominent effects of different engineering parameter on skin friction coefficient $({C}_{fx}{{\mathrm{Re}}_{x}}^{1/2})$, Heat flux $(N{u}_{x}{{\mathrm{Re}}_{x}}^{-1/2})$ and mass flux $(S{h}_{x}{{\mathrm{Re}}_{x}}^{-1/2})$ are numerically computed in Tables 1–3. Table presented the numerical results of $({C}_{fx}{{\mathrm{Re}}_{x}}^{1/2})$. Skin friction force is reduces for the higher value of M while it is boost up for the augmented value of $\beta$. The significant effect of $({\rm{{\rm M}}}),({N}_{r}),({{\rm{{\rm N}}}}_{b}),({{\rm{{\rm N}}}}_{t})$ and $({E}_{c})$ on $(N{u}_{x}{{\mathrm{Re}}_{x}}^{-1/2})$ are computed in Table 2. The higher value of $({\rm{{\rm M}}}),({N}_{r}),({{\rm{{\rm N}}}}_{t})$ and $({E}_{c})$ enhances the heat transfer rate $(N{u}_{x}{{\mathrm{Re}}_{x}}^{-1/2})$ while reverse behaviors is noted for $({{\rm N}}_{b})$. The significant effect of $({\rm{{\rm E}}}),({S}_{c}),({{\rm{{\rm N}}}}_{b})$ and $({{\rm N}}_{t})$ on $(S{h}_{x}{{\mathrm{Re}}_{x}}^{-1/2})$ are computed in Table 3. From Table 3 it is observed that the mass transfer rate varies with augmentation of $({\rm{{\rm E}}}),({S}_{c}),({{\rm{{\rm N}}}}_{t})$ while reduces for $({{\rm{{\rm N}}}}_{t})$.

**Table 1:** Numerical outcome of surface drag force $({C}_{fx}{{\mathrm{Re}}_{x}}^{1/2})$.

M	*β*	M	${C}_{fx}{{\mathrm{Re}}_{x}}^{1/2}$
0.0	0.1	0.1	1.92508
1.0			1.88081
2.0			1.15201
1.0	0.2		1.23321
	0.4		1.81229
	0.6		2.10353
	0.1	0.1	0.85008
		0.2	0.67506
		0.3	0.22358

**Table 2:** Numerical outcome of heat transfer rate $(N{u}_{x}{{\mathrm{Re}}_{x}}^{-1/2})$.

N_*r*_	*M*	*Ec*	*Nb*	*Nt*	$N{u}_{x}{{\mathrm{Re}}_{x}}^{-1/2}$
0.2	0.0	0.2	0.3	0.6	0.76122
0.5					0.89110
0.7					1.10902
0.5	1.0				0.89110
	1.5				0.99921
	2.0				1.31670
	1.0	0.2			1.61151
		0.5			2.01291
		0.7			2.51991
		0.2	0.3		0.90051
			0.5		0.61161
			0.7		0.14890
			0.3	0.6	0.76441
				0.8	0.98762
				1.2	1.12027

**Table 3:** Numerical outcome of mass flux $(S{h}_{x}{{\mathrm{Re}}_{x}}^{-1/2})$.

E	*S*_*c*_	*Nb*	*Nt*	$S{h}_{x}{{\mathrm{Re}}_{x}}^{-1/2}$
0.2	0.5	0.3	0.6	0.56616
0.5				0.57851
0.7				0.67321
0.5	1.0	0.7		0.73172
	1.5			0.88221
	2.0			0.92190
	1.0	0.3		0.92190
		0.5		0.86121
		0.7		0.66163
		0.3	0.6	1.00591
			0.8	1.12198
			1.2	1.428933

## Validation of Results by Comparison

In this section comparison amongst current and published results for validation are presented. Tables 4 and 5 are sketched to validate the exactness of our recent result with published result in literature. Here the comparison of surface drag force $({C}_{fx}{{\mathrm{Re}}_{x}}^{1/2})$, gradient of temperature $(N{u}_{x}{{\mathrm{Re}}_{x}}^{-1/2})$ and concentration gradient $S{h}_{x}{{\mathrm{Re}}_{x}}^{-1/2}$ against M, N_*b*_ and N_*t*_ for varying value are compared with ref. ^32^ and ref. ^48^. Clearly the result is in good agreement.

**Table 4:** Numerical variation of surface drag force $({C}_{fx}{{\mathrm{Re}}_{x}}^{1/2})$ and comparison with ref. ^48^ via various value of M at $\beta =0.1,\,{\rm{M}}=1.0$.

M	ref. ^48^	Current outcome
0.0	0.073135	0.07301
0.5	0.273147	0.27221
0.8	0.636092	0.62660

**Table 5:** Numerical variation of heat and mass transfer rate $(N{u}_{x}{{\mathrm{Re}}_{x}}^{-1/2})$ & $(S{h}_{x}{{\mathrm{Re}}_{x}}^{-1/2})$ and there comparison with ref. ^32^ via various value of ${{\rm{{\rm N}}}}_{b}$ and ${{\rm{{\rm N}}}}_{t}$ at $({\rm{{\rm M}}}=0),\,\beta \to \infty ,{\rm{M}}=1.0,\,{\rm{{\rm P}}}r=10$.

N_*b*_	N_*t*_	Rafique *et al*.^32^		Present results	
		$(N{u}_{x})$	$(S{h}_{x})$	$(N{u}_{x})$	$(S{h}_{x})$
0.1	0.1	0.9524	2.1294	0.95221	2.12881
0.2	0.2	0.3654	2.5152	0.36591	2.51452
0.3	0.3	0.1355	2.6088	0.13501	2.60112
0.4	0.4	0.0495	2.6038	0.04958	2.62381
0.5	0.5	0.0179	2.5731	0.01790	2.57091

## Mean Findings

Here we scrutinize the entropy optimization investigation in electrically conducting Casson nanofluid over nonlinear stretchable surface. Novel behavior of Brownian motion and thermophoresis are also studied. Furthermore physical features activation energy with convective conditions and entropy optimization are studied.The physics of heat and mass transmission are explained and associations were developed for them. The significant observations of present study are given below:

- For greater value of (M) the Lorentz forces enhances which rises the resistive force to the nanofluid motion and in result the velocity ${f}_{\eta }(\eta )$ reduces.
- The increasing values of *M* augmented the heat transfer rate and $\theta (\eta )$ increases.
- Enhancing *β* reduces the shear stress of the fluid and in turn it relax the fluid to move with higher velocity
- We observed enhancement in temperature field $\theta (\eta )$ with higher value of radiation parameter $({N}_{r})$.
- Enhancing $({{\rm{{\rm N}}}}_{b})$ leads to the faster random motion of nanoparticles in fluid flow which displays an extension in thermal boundary layer thickness and augments the temperature of nanofluid more rapidly. A similar configuration is perceived for growing values (Nt) on $\theta (\eta )$
- Concentration $\phi (\eta )$ is increasing function for reaction rate $({\sigma }_{1})$.
- Augmenting activation energy enhances the concentration $\phi (\eta )$ of nanofluid.
- Increasing $({{\rm{{\rm N}}}}_{r})$ increases the emission of thermal radiation and as a results entropy ${{\rm{{\rm N}}}}_{G}(\eta )$ of the nanofluid augmented
- Brinkman number increases the entropy optimization.
- The surface drag force reduces for the higher value of M while it is boost up for the augmented value of *β*.
- The higher value of (M), (*N*_*r*_), (N_*t*_) and (*E*_*c*_) enhances the heat transfer rate $(N{u}_{x}{{\mathrm{Re}}_{x}}^{-1/2})$ while reverse behaviors is noted for $({{\rm{{\rm N}}}}_{b})$.

## References

[1] Casson, N. A flow equation for pigment oil suspensions of the printing ink type. *Rheology of Dispersed System* (1959).

[2] Hayat T, Shehzad SA, Alsaedi A (2012). Soret and Dufour effects on magnetohydrodynamic (MHD) flow of Casson fluid. Applied Mathematics and Mechanics..

[3] Eldabe NTM, Salwa MGE (1995). Heat transfer of MHD non-Newtonian Casson fluid flow between two rotating cylinders. Mechanics and Mechanical Engineering..

[4] Malik MY, Naseer M, Nadeem S, Rehman A (2014). The boundary layer flow of Casson nanofluid over a vertical exponentially stretching cylinder. Applied Nanoscience’s..

[5] Shah, Z., Islam, S., Ayaz, H. & Khan, S. Radiative Heat And Mass Transfer Analysis Of Micropolar Nanofluid Flow Of Casson Fluid Between Two Rotating Parallel Plates With Effects Of Hall Current. *ASME Journal of Heat Transfer*, **2019**, 141/022401-1, 10.1115/1.4040415 (2018).

[6] Kumam Poom, Shah Zahir, Dawar Abdullah, Rasheed Haroon Ur, Islam Saeed (2019). Entropy Generation in MHD Radiative Flow of CNTs Casson Nanofluid in Rotating Channels with Heat Source/Sink. Mathematical Problems in Engineering.

[7] Saravana R, Sailaja M, Reddy R (2018). Effect of aligned magnetic field on Casson fluid flow over a stretched surface of non-uniform thickness. Nonlinear Engineering..

[8] Wong, K. F. V. & Leon, O. D. Applications of nanofluids: current and future. *Adv. Mech. Engng*. 1–11 (2010).

[9] Choi, S. U. S. & Eastman, J. A. Enhancing thermal conductivity of fluids with nanoparticles: The Proceedings of the 1995 ASME InternationalMechanical Engineering Congress and Exposition, San Francisco, USA, ASME, FED 231/MD, 66, 99–105 (1995)

[10] Sheikholeslami M (2019). Heat transfer simulation during charging of nanoparticle enhanced PCM within a channel. Physica A: Statistical Mechanics and its Applications.

[11] Kandasamy R, Hayat T, Obaidat S (2011). Group theory transformation for Soret an Dufour effects on free convective heat and mass transfer with thermophoresis and chemical reaction over a porous stretching surface in the presence of heat source/sink. Nuclear Engineering and Design..

[12] Zubair M (2019). Entropy Generation Optimization in Squeezing Magnetohydrodynamics Flow of Casson Nanofluid with Viscous Dissipation and Joule Heating Effect. Entropy..

[13] Saleem S, Nadeem S, Rashidi MM, Raju CS (2019). An optimal analysis of radiated nanomaterial flow with viscous dissipation and heat source. Microsystem Technologies.

[14] Saleem S, Firdous H, Nadeem S, Khan AU (2019). Convective heat and mass transfer in magneto Walter’s B nanofluid flow induced by a rotating cone. Arabian Journal for Science and Engineering.

[15] Shah Z (2020). Micropolar gold blood nanofluid flow and radiative heat transfer between permeable channels. Computer Methods and Programs in Biomedicine..

[16] Ali I, Aboul-Enein HY, Gupta VK (2009). Microchip-Based Nano Chromatography and Nano Capillary Electrophoresis in Genomics and Proteomics. Chroma.

[17] Shah, Z. *et al*. Influences of electrical MHD and Hall current on squeezing nanofluid flow inside rotating porous plates with viscous and joule dissipationeffects. *J. Therm. Anal. Calorim*, 10.1007/s10973-019-09176-7 (2020)

[18] Sadiq MA, Khan AU, Saleem S, Nadeem S (2019). Numerical simulation of oscillatory oblique stagnation point flow of a magneto micropolar nanofluid. RSC Advances.

[19] Shahzadi I, Suleman S, Saleem S, Nadeem S (2020). Utilization of Cu-nanoparticles as medication agent to reduce atherosclerotic lesions of a bifurcated artery having compliant walls. Computer methods and programs in biomedicine.

[20] Ali I, Al-Othman ZA, Al-Warthan A, Aboul-Enein HY (2014). Recent Trends in Chiral Separations by Nano Liquid Chromatography and Nano Capillary Electrophoresis. Current Chromatography.

[21] Malik MY, Naseer M, Nadeem YS, Rehman A (2014). The boundary layer flow of Casson nanofluid over a vertical exponentially stretching cylinder. Applied Nanosciences..

[22] Hussanan A, Zuki MS, Khan I, Shafie S (2017). Convection heat transfer in micropolar nanofluids with oxide nanoparticles in water, kerosene and engine oil. Journal of Molecular Liquids..

[23] Hussanan A, Trung NT (2019). Heat transfer analysis of sodium Carboxymethyl Cellulose based nanofluid with Tiatania nanoparticles. Journal of Advanced Research in Fluid Mechanics and Thermal Sciences..

[24] Hussanan A, Salleh MZ, Taha TA, Khan I (2018). MHD flow and heat transfer in a Casson fluid over a nonlinearly stretching sheet with Newtonian heating. Heat Transfer Research.

[25] Saleem, S., Awais, M., Nadeem, S., Sandeep, N. & Mustafa, T. Theoritical analysis of upper-convected Maxwell fluid flow with Cattaneo-Christov heat flux model. *Chinese Journal of Physics*, (In Press) (2017).

[26] Sheikholeslami M, Shah Z, Shafi A, Khan I, Itili I (2019). Uniform magnetic force impact on water based nanofluid thermal behavior in a porous enclosure with ellipse shaped obstacle. Scientific report..

[27] Ellahi R, Sait SM, Shehzad N, Mobin N (2019). Numerical simulation and mathematical modeling of electro-osmotic Couette–Poiseuille flow of MHD power-law nanofluid with entropy generation. Symmetry..

[28] Akmal N, Sagheer M, Hussain S, Kamran A (2019). Investigation of free convection in micropolar nanofluid with induced magnetic field Investigation of free convection in micropolar nanofluid with induced magnetic field. European Journal Physics Plus..

[29] Jawad M (2019). Impact of nonlinear thermal radiation and the viscous dissipation effect on the unsteady three-dimensional rotating flow of single-wall carbon nanotubes with aqueous suspensions. Symmetry..

[30] Saeed A (2019). Three-dimensional Casson nanofluid thin film flow over an inclined rotating disk with the impact of heat generation/consumption and thermal radiation. Coatings..

[31] Ashraf, B. M., Hayat, T. & Alsaedi, A. Mixed convection flow of Casson fluid over a stretching sheet with convective boundary conditions and Hall effect. *Boundary Value Problems*. **137** (2017).

[32] Rafique K (2019). Numerical Solution of Casson Nanofluid Flow Over a Non-linear Inclined Surface With Soret and Dufour Effects by Keller-Box Method. Front. Phys..

[33] Bejan A (1996). The entropy generation minimization. Journal of Applied Physics..

[34] Ellahi R, Alamri SA, Basit A, Majeed A (2018). Effects of MHD and slip on heat transfer boundary layer flow over a moving plate based on specific entropy generation. J. Taibah Uni. Sci..

[35] Rashidi MM, Abelman S, Mehr F (2013). Entropy generation in steady MHD flow due to a rotating porous disk in a nanofluid. International Journal of Heat and Mass Transfer..

[36] Atlasa M, Hussain S, Sagheer M (2019). Entropy generation and unsteady Casson fluid flow squeezing between two parallel plates subject to Cattaneo-Christov heat and mass flux. European Journal of Physics Plus..

[37] Afridi MI, Qasim M, Khan I (2018). Entropy generation minimization in MHD boundary layer flow over a slendering stretching sheet in the presence of frictional and Joule heating. Journal of Korean Phys. Soc..

[38] Noreen S, Abbas A, Hussanan A (2019). Entropy generation via Ohmic heating and Hall current in Peristaltically-Flowing Carreau fluid. Entropy..

[39] Abolbashari MH, Freidoonimehr N, Nazari F, Rashidi MM (2014). Study on entropy generation of a MHD nanofluid flow towards a stretching surface. Powder Technolgy..

[40] Sheikholeslami M, Ganji DD (2015). The entropy generation of a nanofluid. Physica A..

[41] Ellahi, R., Raza, M. & Akbar, N. S. Study of peristaltic flow of nanofluid with entropy generation in a porous medium. *Journal of Porous Media*. **20**(5) (2017).

[42] Ellahi R, Hassan M, Zeeshan A, Khan A (2016). The shape effects of nanoparticles suspended in HFE-7100 over wedge with entropy generation and mixed convection. Applied Nanoscience..

[43] Feroz N, Shah Z, Islam S, Alzahrani EO, Khan W (2019). Entropy Generation of Carbon Nanotubes Flow in a Rotating Channel with Hall and Ion-Slip Effect Using Effective Thermal Conductivity Model. Entropy.

[44] Dawar A, Shah Z, Khan W, Idrees M, Islam S (2019). Unsteady squeezing flow of MHD CNTS nanofluid in rotating channels with Entropy generation and viscous Dissipation, Advances in. Mechanical Engineering.

[45] Daniel, Y. S., Aziz, Z. A., Ismail, Z. & Salah, F. Entropy Analysis of Unsteady Magnetohydrodynamic Nanofluid over Stretching Sheet with Electric Field. *International Journal for Multiscale Computational Engineering*. **15**(6) (2017)

[46] Ali I, Alharbi OML (2016). Marsin Sanagi., Nano-capillary electrophoresis for environmental analysisEnviron. Chem. Lett..

[47] Sheikholeslami M (2019). New computational approach for exergy and entropy analysis of nanofluid under the impact of Lorentz force through a porous media. Computer Methods in Applied Mechanics and Engineering.

[48] Hayat T, Riaz R, Aziz A, Alsaedi A (2019). Analysis of entropy generation for MHD flow of third grade nanofluid over a nonlinear stretching surface embedded in a porous medium. Phys. Scr..

[49] Reddy GJ, Kethireddy B, Kumar M, Hoque MM (2018). A molecular Dynamics Study on transient non-Newtonian MHD Casson fluid flow dispersion over a radiative vertical cylinder with entropy heat generation. Journal of Molecular Liquids..

[50] Alothman, Z. A., & Ali, I. Nano capillary electrophoresis in microchips: A need of the present century. *Journal of Liquid Chromatography & Related Technologies* **3****4**(14), 1295–1325.

